# Eliminating Medicare bad debt payments: are critical access and rural hospitals at risk?

**DOI:** 10.1093/haschl/qxaf220

**Published:** 2025-11-28

**Authors:** Dunc Williams, Ganisher Davlyatov, John R Bowblis, Robert Tyler Braun

**Affiliations:** Healthcare Financial Management, College of Health Professions, Department of Health Care Leadership and Management, Medical University of South Carolina, Charleston, SC 29425, USA; Department of Health Administration and Policy, Hudson College of Public Health, University of Oklahoma Health Campus, Oklahoma City, OK 73104, USA; Department of Economics and Scripps Gerontology Center, Miami University, Oxford, OH 45056, USA; Cornell Health Policy Center, Cornell University, New York, NY 10021, USA

**Keywords:** healthcare finance, healthcare accounting, cost report, Medicare allowable bad debt, hospital policy

## Abstract

**Introduction:**

While Medicare Allowable Bad Debt (MBD), defined as unpaid patient financial obligations Medicare partially reimburses to hospitals, represents only 0.12% of patient revenue, policymakers and executives should note the $1.7 billion reimbursed in 2022 affects hospitals of different types in various ways (see Appendix 1). The recent passage of the One Big Beautiful Bill Act did not eliminate MBD, but elimination has been proposed, supporting a need to understand what elimination could do to hospitals.

**Methods:**

Using Medicare Cost Reports, we conducted a retrospective, longitudinal analysis of short-term general acute-care hospitals in 2022.Total margin was evaluated with and without MBD by Critical Access Hospital (CAH), rurality, state, and Hospital Referral Region.

**Results:**

Elimination of this reimbursement would have impacted many hospitals; though findings highlight more adverse impacts on CAHs (accounting for a 0.3% point [PP] reduction in total margin), other rural hospitals (a 0.25PP reduction), states like Nevada (a 0.48PP reduction), and certain HRRs around the Appalachia region and parts of Texas.

**Conclusion:**

Elimination of MBD may further jeopardize financial solvency for some rural hospitals that provide access to acute care across America's vast (mostly rural) land mass, particularly in rural and underserved communities.

## Introduction

In 2022, it was estimated that Medicare paid hospitals only 82 cents for every dollar of care delivered, creating nearly $100 billion in annual underpayments and contributing to two-thirds of all hospitals reporting negative Medicare Margins.^1^ While some question whether Medicare truly underpays hospitals,^2-4^ persistent negative profit margins and seemingly technical reimbursement rules can determine a hospital's survival. One rule under scrutiny often at the center of deficit-reduction discussions is Medicare's “allowable bad debt”—unpaid deductibles and coinsurance from beneficiaries. Under current regulations, the Centers for Medicare & Medicaid Services (CMS) reimburses hospitals 65% for these bed debt losses.^5^

While CMS reimbursement of Medicare bad debt has never fallen below 55%, this payment has long been a target in federal budget negotiations, with renewed efforts underway to reduce or eliminate it. The idea persists in policy circles because of the federal savings it offers. The recent “Fair Care Act” proposed reducing reimbursement from 65% to 25%.^6^ For example, the Congressional Budget Office estimates that ending Medicare bad debt reimbursement entirely would eliminate federal outlays by about $54 billion over ten years.^7^ Opponents state these savings, however, could come at the cost of further straining hospital finances; especially for rural facilities and those dependent on Medicare, where even small losses in reimbursement can threaten continued operation.

Earlier studies have suggested that cutting Medicare allowable bad debt (MBD) payments would erode hospital margins, but the evidence base is dated and limited in scope. For instance, a 2007 brief modeled the impact of repeal only for a subset of rural hospitals,^8^ while other analyses combined bad-debt cuts with unrelated payment changes, making specific effects difficult to isolate.^9^ This is often because analyses bundle proposed reductions to bad debt reimbursement with other policy levers, such as modifications to Disproportionate Share Hospital payments, or they did not distinguish Medicare-specific losses from a provider's total bad debt and charity care portfolio.^10^ Other studies^11,12^ have estimated total dollars at risk differentiating the financial impact across the continuum of care, but not separating findings for acute care (inpatient and outpatient services), free-standing inpatient rehabilitation facilities, long-term care hospitals, and integrated post-acute care units (eg, skilled nursing and home health).

Additional previous analyses predate the financial shifts triggered by the COVID-19 pandemic. Since 2020, hospitals have faced persistently high inflation, higher labor costs, pharmaceutical price increases, and ongoing supply chain challenges.^13^ Many hospitals became reliant on costly contract labor to fill staffing gaps, straining operating budgets. At the same time, the rise of Medicare Advantage—now covering more than half of all Medicare beneficiaries—has shifted hospitals' payer mix toward plans that typically reimburse less and deny more claims than traditional Medicare.^14,15^ One recent important study evaluating Medicare bad debt highlighted differences in “distributional consequences” of eliminating Medicare's bad debt reimbursement, calling for further understanding of such a policy change that are “inadequately understood”.^16^ This research builds upon that work by calculating the impact of such an elimination on profits, and doing so by hospital type and across geographical differences.

Higher structural costs and tighter reimbursement make the potential loss of bad debt reimbursement far more consequential than earlier estimates suggest. An updated understanding is needed—particularly of how rural vs urban hospitals, critical access hospitals (CAHs), and specific regions would be affected if this reimbursement were eliminated.

Using hospital cost reports from 2022, this study addresses three key research questions: (1) How would financial margins of U.S. acute-care hospitals have changed if current Medicare bad debt payments were fully eliminated? (2) What type of hospitals would shift from positive to negative margins under these scenarios? and (3) Which regions of the U.S. would be most impacted if bad debt payments were eliminated?

## Study data and methods

### Study design and data sources

We conducted a retrospective, longitudinal analysis of short-term general acute-care hospitals in 2022. Hospital financial and operational data were extracted from the CMS Hospital Cost Report Information System files, “cost reports.”^17,18^ Hospital-level characteristics were sourced from the CMS Hospital Cost Report Information System (1) combined with market population data from the American Community Survey and system affiliation characteristics from the Agency for Healthcare Research and Quality (AHRQ) Compendium of U.S. Health Systems, American Hospital Association (AHA) Annual Survey, and the Provider Enrollment, Chain, and Ownership System (PECOS) Hospital Change of Ownership (CHOW).

### Sample

The study includes all short-term general acute care U.S. hospitals that that filed a complete Medicare cost report in 2022. Exclusions were applied to hospitals with incomplete financial data (eg, less than 360 days in a reporting period) or those not classified as general acute care facilities. The final analytic sample had 4106 unique hospitals.

### Variables

Our key variable of interest was total margin, with and without Medicare allowed bad debt payments. Total margin measures the overall profitability of a hospital's performance and is defined as converting net income (exclusive of any COVID-19 relief funds) divided by revenue into a percentage. The advantage of total margin is it allows for comparison of hospitals of various sizes. A higher total margin indicates better financial health, while negative margins indicate the hospital is losing money and is less sustainable.

When calculating total margin with bad debt payments, we used the net income reported by the hospital and we removed COVID-19 reported revenues. To calculate total margin without bad debt payments, we subtracted MBD payments from net income. Medicare allowable bad debt payment is defined as only the reimbursable portion of Medicare bad debt, as reported by hospitals on Cost Report Worksheet S-10, Row 27, Column 1, which reflects net reimbursement after accounting for the 65% eligible reimbursement. Rurality was defined according to the criteria specified by the Federal Office of Rural Health Policy.^19^

### Statistical analyses

Our main analysis compared the total margin of hospitals with and without Medicare allowed bad debt payments. We compared these total margins by type of hospital, rurality, state, and Hospital Referral Region (HRR). These descriptive statistics were calculated after winsorizing all continuous variables at the first and 99th percentiles to limit the influence of extreme outliers (3, 4). Group differences were assessed with independent samples t-tests for continuous variables and Pearson chi-square tests for categorical variables, with α = 0.05 defining statistical significance. All analyses were conducted in Stata 18 (StataCorp, College Station, TX). Geographical distance to the nearest hospital was calculated as the straight-line Haversine distance of the latitude and longitude between ZIP centroids of hospitals.

### Limitations

Medicare Cost Reports are a widely used source of hospital financial information. All hospitals must file cost reports annually to receive Medicare reimbursement. However, because hospitals self-report data, Medicare Cost Reports can exhibit inconsistencies.^20^ Nevertheless, the availability of financial data for all hospitals and other health care providers receiving CMS reimbursement, combined with the frequent use by researchers and finance professionals, makes Medicare Cost Reports a commonly accepted source.^21,22^

We evaluated a cross-section of data for 2022, yet average profitability changes over years. For hospitals in which we found profitability signs would have changed from positive to negative in 2022 without Medicare bad debt payments, we conducted an unreported sensitivity analysis and showed similar distributions of hospitals that would have similarly reported profitability sign changes across CAHs, other rural, and urban hospitals.

Additionally, the health system affiliation variable we constructed is limited to only hospitals recorded as part of a system from the AHRQ Compendium (for years 2016-2022), the AHA Annual Survey (for which we only had access to data from years 2000-2021), or the PECOS CHOW. Hospitals within a system that were not recorded by any of these datasets within those years were not recognized as being in a system.

## Results

Table 1 shows that in 2022, there were 4106 short-term general acute care hospitals in our analytic sample (1215 CAH, 844 rural, 2047 urban). Medicare allowed bad debt averaged $419 thousand per hospital and amounted to approximately 0.3% of net patient revenues (NPR). Medicare bad debt averaged $650 000 or 0.2% of NPR for urban hospitals, $154 000 or 0.4% of NPR for CAHs, and $244 000 or 0.3% of NPR for rural hospitals.

**Table 1.** Unadjusted organizational, operational, and financial characteristics by rurality, 2022.

Unadjusted hospital characteristics—CAH, rural, urban, total, hospitals with margin changes—2022.					
	CAH	Rural	Urban	Total	Hospitals whose total margin would have converted negative without this reimbursement
N	1215 (29.6%)	844 (20.6%)	2047 (49.9%)	4106 (100.0%)	42 (1.0%)
Total Medicare Allowable Bad Debt	153 624 (227 307)	243 753 (292 788)	649 688 (646 718)	419 457 (543 176)	416 916 (517 102)
Medicare Allowable Bad Debt as a Percent of Net Patient Revenue (%)	0.423 (0.435)	0.267 (0.299)	0.184 (0.168)	0.272 (0.315)	0.507 (0.517)
Total Margin, COVID Funds Removed (%)	1.537 (10.605)	−1.523 (12.136)	1.777 (12.397)	1.034 (11.903)	0.220 (0.263)
Total Margin without COVID Funds—No Medicare Allowable Bad Debt	1.230 (10.527)	−1.773 (12.179)	1.606 (12.443)	0.806 (11.918)	−0.245 (0.320)
Miles to Nearest Hospital	22.350 (12.732)	19.250 (9.883)	5.721 (8.405)	13.384 (12.785)	15.404 (9.576)
					CAH: 21 (50.0%)
					Rural: 7 (16.7%)
					Urban: 14 (33.3%)

Source: Authors' analysis of Cost Reports from 2011-2022.

All between-group differences were statistically significant at *P* < .001. One-way ANOVA tests with Bonferroni pairwise comparisons across hospital types were also significant at *P* < .001 for all variables.

**Figure 1.**
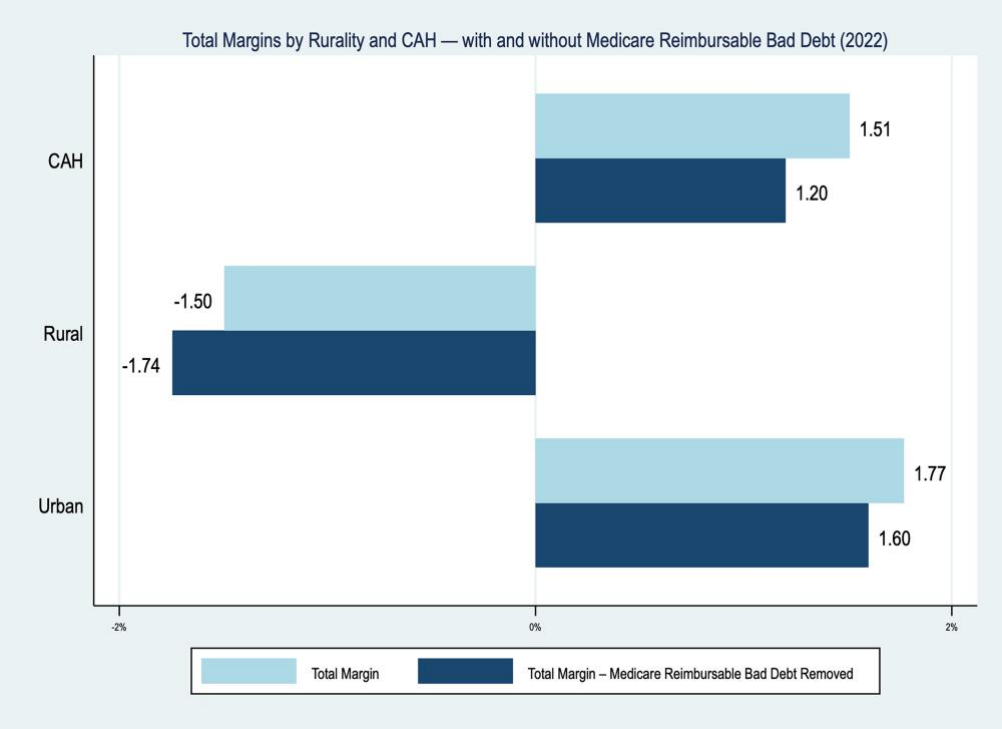
Total margins by rurality and CAH - with and without Medicare reimbursable bad debt (2022). Source: Authors' analysis of cost reports from 2011-2022.

Average hospital total margin was 1.0% without COVID-19 relief funds; eliminating Medicare bad debt payments would decrease the average total margin to 0.8%. However, there was significant variation in profitability by type of hospital with and without the elimination of Medicare bad debt payments. With Medicare bad debt payments, the average urban and CAH were profitable (1.8% and 1.5% total margin, respectively), while the average other rural hospital was not profitable (−1.5% total margin). If Medicare bad debt payments were to be eliminated (Figure 1) urban hospitals and CAHs would have small decreases of 0.2 (1.6% total margin) and 0.3 (1.2% total margin) percentage point decreases, respectively. However, of the 844 other rural hospitals in our sample, the elimination of Medicare bad debt payments would decrease rural hospital total margins from −1.5% to −1.8%. Rural hospitals were, on average, 19 miles from the next closest hospital; CAHs averaged 22 miles.

The elimination of Medicare bad debt payments also causes 42 hospitals to go from having a positive total margin of 0.2% on average, to a negative total margin (−0.2%, Table 1). This means that 42 profitable hospitals would have been unprofitable without MBD. Of those 42 hospitals, 21 (50%) are CAHs, 14 (33%) are urban hospitals, and 7 (17%) are rural hospitals (reflected in the last three rows of data in Table 1). For those 42 hospitals, the next closest hospital is an average of 15.4 miles away.

The impact of eliminating Medicare bad debt payments is not geographically uniform across the country. Rural hospitals tend to be located in the Midwest (26.2%) and the South (53.8%), and the 42 hospitals that would go from a positive to a negative margin if Medicare bed debt payments were eliminated are concentrated in the Midwest (45.2%) and South (26.2%) (see Appendix 2).

**Figure 2.**
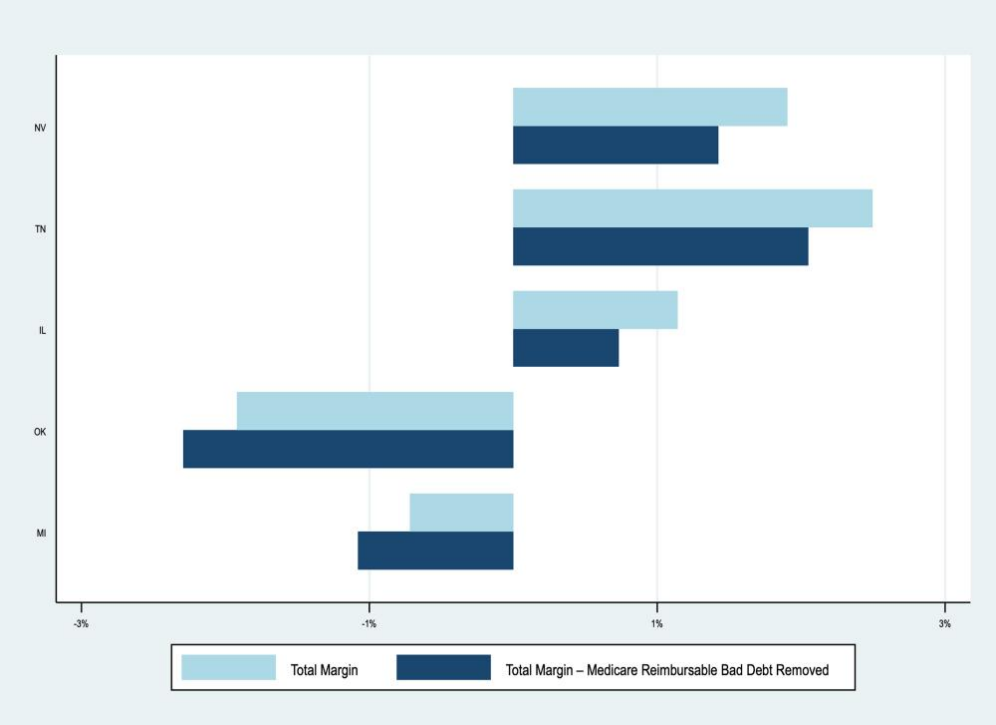
Total margins by state - with and without Medicare reimbursable bad debt (2022). Source: Authors' analysis of cost reports from 2011-2022.

Hospitals in some states would face larger declines in total margin if Medicare bad debt payments were eliminated. Figure 2 shows total margins with and without these payments for the five most affected states, ranked in descending order (see Appendix 3 for the full list). In Nevada—the most affected—hospitals averaged 1.9% total margin in 2022, which would have dropped to 1.4% without bad debt reimbursement (a 0.48% point decrease). In total, 22 states would average negative total margins without this support, and in one state (Wyoming), the average hospital would shift from a positive to a negative total margin.

**Figure 3.**
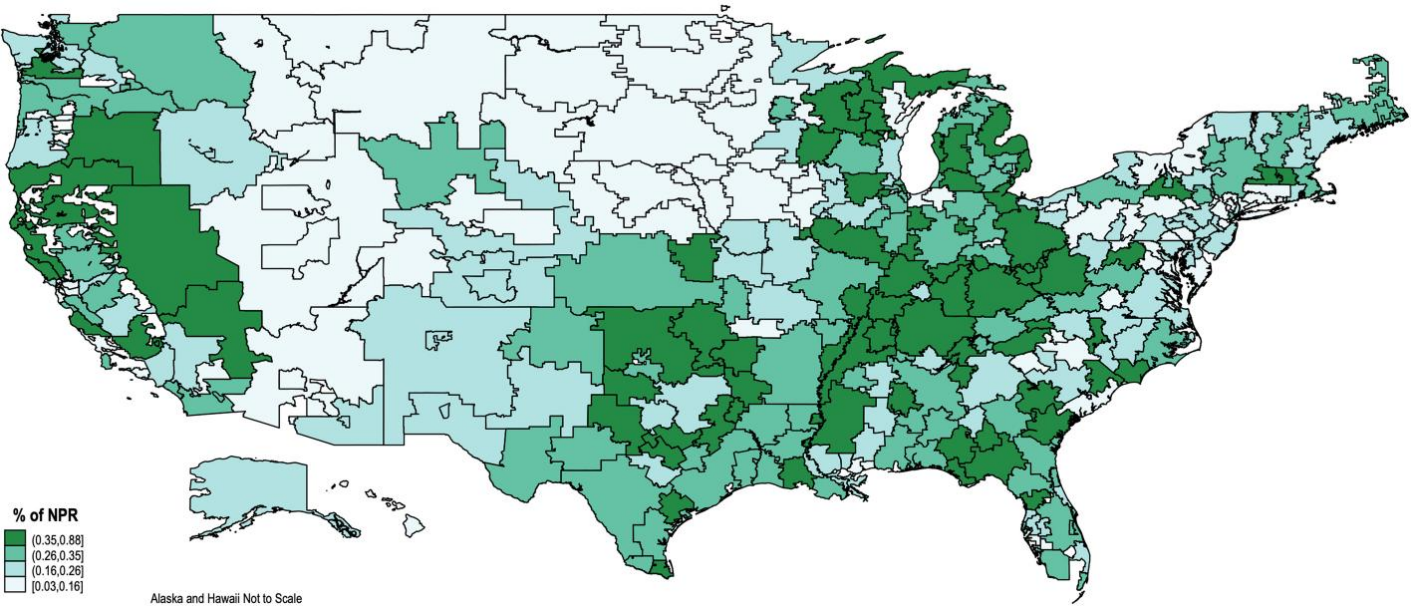
Medicare reimbursable bad debt as a percent of net patient revenue (2022). Source: Authors' analysis of cost reports from 2011-2022.

To examine geographic variation differently than at the state level, Figure 3 illustrates the proportion of NPR that is from Medicare bad debt payments by hospital referral region. Darker areas, which show Medicare bad debt payments comprise a larger share of revenues are located in the southern Appalachia area, parts of northern/central Texas, and Nevada. Hospitals in these areas would have the greatest impact if Medicare bad debt payments were eliminated.

## Discussion and conclusion

In 2022, the average acute-care hospital reported $419 thousand in MBD. Eliminating these payments would reduce the average hospital's profit margin from 1.0% to 0.8%. While the aggregate impact may appear modest, this average masks significant variation as certain hospitals and regions would face far greater financial strain if these payments disappeared.

Our results indicated that Medicare bad debt payments make up the largest proportion of revenues for CAHs (0.4%) followed by rural hospitals that are not CAHs (0.3%) and urban hospitals (0.2%). This might suggest that CAHs would be the most affected by the elimination of Medicare bad debt payments, but our results do not find this. CAHs are located in isolated areas, have under 25 beds, and because of their importance for providing access to these areas, are reimbursed 101% of their allowable cost of care by Medicare.^23^ This makes CAHs profitable, and eliminating Medicare bad debt payments for CAHs still allows them to be profitable on average.

However, this is not the case for rural hospitals that are not CAHs. Other rural hospitals are reimbursed under Medicare's prospective payment system. Additionally, many of these hospitals are in communities which have older populations,^24^ and thus may have fewer privately-insured patients to subsidize lower payments from Medicare and Medicaid. As our results show, the average rural hospital has a negative profit margin even with Medicare bad debt payments.

While Medicare bad debt payments were a relatively small proportion of total revenue for the average hospital, eliminating those payments would only further erode the financial condition of these rural hospitals. Our findings highlight that eliminating Medicare bad debt payments for rural hospitals may only accelerate hospital closures and service reductions in rural communities. Since 2010, over 150 rural hospitals have closed,^25^ and many have eliminated or reduced important services such as obstetrics. The elimination of Medicare bad debt payments could continue this trend, and is likely to hit rural communities in the Midwest and South the hardest.

Rural and CAHs already experience significant distance to the nearest hospital (19 and 22 miles, respectively). Closures or the elimination of certain services would only increase that distance, delaying access to care, especially in emergency situations. The states where the average total margin would be most negatively impacted by elimination of Medicare bad debt payments were Nevada, Tennessee, Illinois, Oklahoma, and Michigan. Some of the most impacted hospital referral areas were around the southern Appalachia area, parts of Texas, and Nevada. Many of these areas already have access issues and poor health outcomes.^26,27^ Without offsetting such an elimination of Medicare bad debt reimbursement with a different reimbursement lever, such as MEDPAC's proposed “Medicare Safety-Net Index,”^28^ profits will be negatively and disproportionately impacted across CAHs, other rural hospitals, and various geographic areas.

In conclusion, our analysis highlights the need for examining policies in a more nuanced manner. Examining Medicare bad debt payment elimination on a national-level looks like a policy that would save money and have limited impact on the average hospital. However, our analysis shows that elimination of Medicare bad debt payments without accounting for these differences could adversely impact access to acute care hospitals, particularly in rural and underserved communities.

## Supplementary Material

qxaf220_Supplementary_Data
