# Supplementary material for: Eliminating Medicare bad debt payments: are critical access and rural hospitals at risk?
Source: Health Aff Sch. 2025 Nov 28;3(11):qxaf220. doi: 10.1093/haschl/qxaf220 (PMC12661523; doi:10.1093/haschl/qxaf220)
Supplement: qxaf220_Supplementary_Data [file qxaf220_supplementary_data.zip › Mcare - Appendix 11_12_25.docx]

# Appendix 1: Gross Charges, Allowances, NPR, Bad Debt, and Medicare Allowable Bad Debt (2022)

| **Gross Charges to Net Patient Revenue; Medicare Allowable Bad Debt** | | | | |
| --- | --- | --- | --- | --- |
| **Explanation with 2022 Hospital Cost Report Data** | | | | |
| ***Inclusive of all hospitals that were in US states, not VA Federal Government, and not IHS (which is, a larger population that our cleaned data set).** | | | | |
| **Financial Indicator** | **2022 Amount** | **% of Gross Charges (Billed, Not Collected)** | **Definition** | **Source (All Cost Report, Wkst-Row-Column)** |
| Gross Charges (Billed, Not Collected) | $5591.2 billion dollars |  | Total charges billed (not collected) | G3,1,1; which is from G2Pt1,28,3 |
| Contractual Allowances and Discounts | $4228.0 billion dollars | 75.62% | Discounts between gross charges (billed) and net patient revenues (collected). CMS defines these as: provision for bad debt, contractual adjustments, charity discounts, teaching allowances, policy discounts, administrative adjustments, implicit price concessions, and other deductions from revenue. | G3,2,1 |
| Net Patient Revenue | $1358.8 billion dollars | 24.30% | Net patient revenue is what was collected after contractual allowances, bad debt, and charity/indigent care. | G3,2,3 |
|  |  |  |  |  |
| Provision for Bad Debt (Gross Charges, Total, with M'care Reimburse) | $50.5 billion dollars | 3.72% | Bad debt, inclusive of Medicare allowable bad debt. | S-10,26,1 |
| Medicare Allowable Bad Debt (Net, not Gross) | $1.7 billion dollars | 0.12% | Medicare allowable portion of bad debt (65% of allowable for most hospitals, varied over time) | S-10,27,1 |
| **Note: For net patient revenue, if we subtract gross charges from contractual allowances, the calculation differs slightly because it would be subtracting means, not the underlying values.* | | | | |

# Appendix 2: Additional Unadjusted Organizational, Operational, and Financial Characteristics by Rurality, 2022

| Table 1: Unadjusted Hospital Characteristics – CAH, Rural, Urban, Total, Hospitals with Margin Changes – 2022 | | | | | | |
| --- | --- | --- | --- | --- | --- | --- |
|  | **CAH** | | **Rural** | **Urban** | **Total** | **Hospitals whose Total Margin Would have Converted Negative without this Reimbursement** |
| **N** | 1,215 (29.6%) | | 844  (20.6%) | 2,047 (49.9%) | 4,106 (100.0%) | 42 (  1.0%) |
| **Hospital Beds - Total** | 22.094 (5.805) | | 75.969 (63.428) | 255.761 (202.369) | 149.640 (181.078) | 90.145 (119.978) |
| **Region** | |  | | | | |
| **Northeast** | 72 (5.9%) | | 83 (9.8%) | 359 (17.5%) | 514 (12.5%) | 3  (7.1%) |
| **Midwest** | 544 (44.8%) | | 221 (26.2%) | 461 (22.5%) | 1,226 (29.9%) | 19  (45.2%) |
| **South** | 350 (28.8%) | | 454 (53.8%) | 776 (37.9%) | 1,580 (38.5%) | 11  (26.2%) |
| **West** | 249 (20.5%) | | 86 (10.2%) | 451 (22.0%) | 786 (19.1%) | 9  (21.4%) |
| **Ownership Type** |  | |  |  |  |  |
| **Not for Profit** | 706 (58.1%) | | 498 (59.0%) | 1,342 (65.6%) | 2,546 (62.0%) | 25  (59.5%) |
| **For Profit** | 58 (4.8%) | | 145 (17.2%) | 498 (24.3%) | 701 (17.1%) | 6  (14.3%) |
| **Government - Non-Federal** | 451 (37.1%) | | 201 (23.8%) | 207 (10.1%) | 859 (20.9%) | 11  (26.2%) |
| **Not Part of a Health** | 362 (29.8%) | | 106 (12.6%) | 65 (3.2%) | 533 (13.0%) | 13  (31.0%) |
| **Part of a Health**  **System** | 853 (70.2%) | | 738 (87.4%) | 1,982 (96.8%) | 3,573 (87.0%) | 29  (69.0%) |
| **Miles to Nearest Hospital** | 22.350 (12.732) | | 19.250 (9.883) | 5.721 (8.405) | 13.384 (12.785) | 15.404 (9.576) |

# Appendix 3: Total Margin by State (All) – with and without Medicare Allowable Bad Debt (2022)

| State | Total Margin | Total Margin – Medicare Allowable Bad Debt Removed | Difference |
| --- | --- | --- | --- |
| NV | 1.904 (.) | 1.425 (.) | 0.480 (.) |
| TN | 2.717 (.) | 2.269 (.) | 0.448 (.) |
| IL | 1.142 (.) | 0.733 (.) | 0.409 (.) |
| OK | -1.866 (.) | -2.228 (.) | 0.362 (.) |
| MI | -0.730 (.) | -1.091 (.) | 0.361 (.) |
| GA | 0.985 (.) | 0.640 (.) | 0.345 (.) |
| MA | -2.540 (.) | -2.880 (.) | 0.340 (.) |
| KY | 3.129 (.) | 2.800 (.) | 0.329 (.) |
| WI | 4.163 (.) | 3.837 (.) | 0.326 (.) |
| KS | -4.463 (.) | -4.784 (.) | 0.321 (.) |
| IN | 3.536 (.) | 3.222 (.) | 0.314 (.) |
| WV | 0.719 (.) | 0.415 (.) | 0.304 (.) |
| CA | -1.340 (.) | -1.642 (.) | 0.302 (.) |
| OH | 2.098 (.) | 1.798 (.) | 0.300 (.) |
| AL | -2.844 (.) | -3.142 (.) | 0.298 (.) |
| MS | -3.858 (.) | -4.139 (.) | 0.281 (.) |
| LA | 3.267 (.) | 3.000 (.) | 0.267 (.) |
| ME | -1.184 (.) | -1.447 (.) | 0.263 (.) |
| WA | -3.262 (.) | -3.518 (.) | 0.256 (.) |
| MD | -2.613 (.) | -2.866 (.) | 0.253 (.) |
| OR | 0.091 (.) | -0.160 (.) | 0.251 (.) |
| TX | 6.184 (.) | 5.941 (.) | 0.243 (.) |
| FL | 6.798 (.) | 6.558 (.) | 0.240 (.) |
| AR | -3.968 (.) | -4.200 (.) | 0.232 (.) |
| NC | 1.630 (.) | 1.404 (.) | 0.226 (.) |
| MO | 0.233 (.) | 0.018 (.) | 0.215 (.) |
| WY | 0.097 (.) | -0.117 (.) | 0.213 (.) |
| NH | 2.642 (.) | 2.433 (.) | 0.209 (.) |
| VT | -3.976 (.) | -4.179 (.) | 0.203 (.) |
| SC | 2.605 (.) | 2.414 (.) | 0.192 (.) |
| VA | 5.690 (.) | 5.499 (.) | 0.191 (.) |
| AK | 3.131 (.) | 2.954 (.) | 0.177 (.) |
| NJ | 0.197 (.) | 0.027 (.) | 0.170 (.) |
| RI | -4.433 (.) | -4.594 (.) | 0.161 (.) |
| PA | -1.353 (.) | -1.501 (.) | 0.148 (.) |
| NM | -0.768 (.) | -0.915 (.) | 0.146 (.) |
| DE | -2.973 (.) | -3.117 (.) | 0.144 (.) |
| ID | 1.016 (.) | 0.876 (.) | 0.140 (.) |
| NY | -4.584 (.) | -4.724 (.) | 0.140 (.) |
| CO | 2.070 (.) | 1.945 (.) | 0.125 (.) |
| AZ | 2.026 (.) | 1.922 (.) | 0.104 (.) |
| ND | -0.660 (.) | -0.758 (.) | 0.098 (.) |
| IA | 1.263 (.) | 1.170 (.) | 0.093 (.) |
| MT | -1.248 (.) | -1.332 (.) | 0.084 (.) |
| NE | 1.582 (.) | 1.498 (.) | 0.084 (.) |
| MN | 0.857 (.) | 0.795 (.) | 0.062 (.) |
| SD | 3.871 (.) | 3.809 (.) | 0.062 (.) |
| UT | 12.711 (.) | 12.656 (.) | 0.055 (.) |
| CT | -2.063 (.) | -2.109 (.) | 0.046 (.) |
| HI | -0.638 (.) | -0.684 (.) | 0.045 (.) |
| DC | 1.500 (.) | 1.476 (.) | 0.023 (.) |
